# Beneficial effect of estrogen on nigrostriatal dopaminergic neurons in drug-naïve postmenopausal Parkinson’s disease

**DOI:** 10.1038/s41598-019-47026-6

**Published:** 2019-07-19

**Authors:** Yang Hyun Lee, Jungho Cha, Seok Jong Chung, Han Soo Yoo, Young H. Sohn, Byoung Seok Ye, Phil Hyu Lee

**Affiliations:** 1 Department of Neurology, Yonsei University College of Medicine, Seoul, South Korea; 2 Memory and Aging Center, Department of Neurology, University of California San Francisco, San Francisco, CA USA; 3 Severance Biomedical Science Institute, Yonsei University College of Medicine, Seoul, South Korea

**Keywords:** Parkinson's disease, Parkinson's disease

## Abstract

This study aimed to investigate the potential beneficial effects of estrogen on nigrostriatal dopaminergic neuron degeneration in postmenopausal drug-naïve Parkinson’s disease (PD). Based on the ratio of lifetime estrogen exposure length to the total length of the estrogen exposure and deprivation period, postmenopausal women with drug-naïve PD were divided into low (n = 31) and high (n = 31) estrogen exposure ratio groups. We performed a comparative analysis of the striatal dopamine transporter (DAT) availability between the two groups. Additionally, we evaluated the longitudinal change in the levodopa equivalent dose per month using a linear mixed model. The motor symptoms were more severe in the low estrogen exposure ratio group than in the high estrogen exposure ratio group (*P* = 0.016). PD patients in the two groups had significantly lower DAT availability on all striatal sub-regions except for ventral striatum than did age- and sex-matched normal controls. When comparing the two groups, PD patients in the low estrogen exposure ratio group exhibited significantly lower DAT availability in the posterior putamen (*P* = 0.024) and in the ventral putamen (*P* = 0.036) than those in the high estrogen exposure ratio group. The estimated monthly levodopa equivalent dose changes were 10.9 in the low estrogen exposure ratio group and 7.1 in the high estrogen exposure ratio group with a significant interaction between the two groups (*P* = 0.001). These *in vivo* data provide indirect evidence showing that estrogen may elicit a beneficial effect on nigrostriatal dopamine neurons in PD.

## Introduction

Epidemiologic studies have revealed that the prevalence of Parkinson’s disease (PD) is 1.5–2-fold lower in women than in men^1,2^, suggesting a possible protective influence of estrogen in predilection to the disease. Experimental evidences suggest that estradiol exerts neuroprotective effects on dopaminergic neurons and promotes dopaminergic activity in the striatum^3,4^. Observational studies have indicated that increased length of estrogen exposure period was correlated with less severe PD symptoms and that the risk of developing PD was increased in women with experience of surgical menopause^5,6^. These data suggest that estrogen may have a protective effect on dopaminergic neurons in PD.

However, several studies have demonstrated that the incidence of PD in women appears to vary depending on the formulation and dose of hormone therapy, timing and length of dosing period, and history of surgical or natural menopause^7,8^. Furthermore, one animal study reported that long-term estrogen deprivation by prolonged ovariectomy in primates might be an important determinant in maintaining nigrostriatal dopamine neurons^9^. This finding suggests that not only the total length of lifetime estrogen exposure, but also the total period of estrogen deprivation might be crucial factors to modulate the loss of dopaminergic neurons.

Until now, the potential beneficial effects of estrogen on nigrostriatal dopaminergic degeneration in patients with PD have not been investigated using *in vivo* neuroimaging. In this study, according to the ratio of estrogen exposure length to the total length of the estrogen exposure and deprivation period (E_ratio_), we performed a comparative analysis of the extent of presynaptic dopaminergic depletion by quantitatively measuring N-(3-[18F]fluoropropyl)-2β-carbon ethoxy-3β-(4-iodophenyl) nortropane (18F-FP-CIT) positron emission tomography (PET) in postmenopausal women with drug-naïve PD. Additionally, we evaluated the influence of E_ratio_ on the longitudinal requirement of dopaminergic medications in these patients.

## Methods

### Subjects

**Figure 1:**
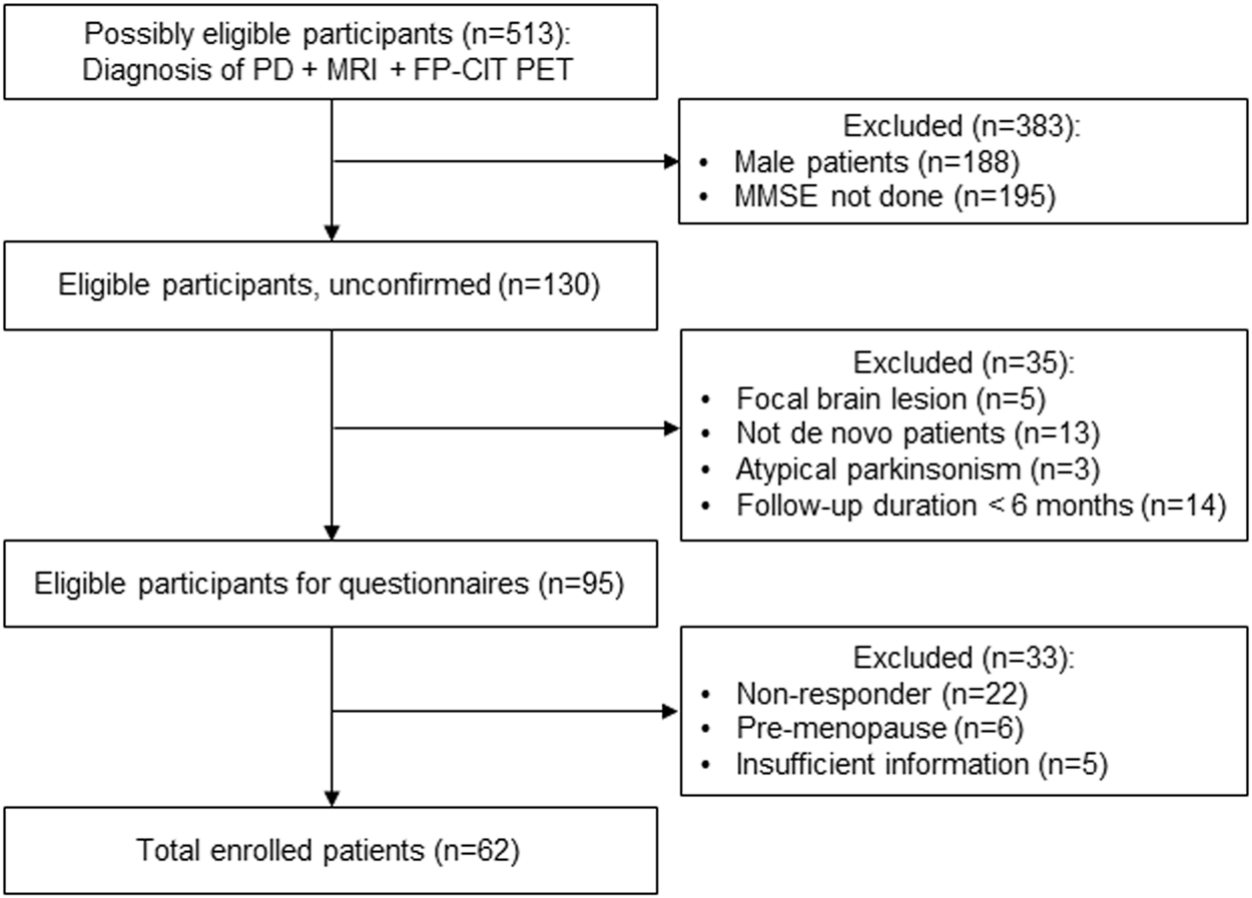
Study flow chart.

Subjects who visited the movement disorders clinic at Severance Hospital, Yonsei University from May 2010 to March 2018 were recruited from the database of the Yonsei Parkinson Center. The details of the enrollment of the study participants are illustrated in Fig. 1. Both ^18^F-FP-CIT PET scan and brain magnetic resonance imaging (MRI) were undergone within two months after initial visit to the clinic and before the start of dopaminergic medications. PD was diagnosed according to the clinical diagnostic criteria of the UK PD Society Brain Bank, and each subject exhibited decreased dopamine transporter (DAT) availability in the posterior putamen (PP) on the ^18^F-FP-CIT PET scans^10^. A total of 62 postmenopausal women out of 513 patients with drug-naïve PD were enrolled in this study.

To assess the severity of parkinsonian motor symptoms, the Unified Parkinson Disease Rating Scale Part III (UPDRS-III) was used. We calculated tremor and non-tremor scores for each patient according to the previously described method and classified patients into three subtypes: tremor dominant, akinetic-rigid, and mixed^11^. General cognition was scored using the K-MMSE score. The UPDRS-III and K-MMSE were measured at the initial visit to the clinic. Olfactory function was assessed with the Cross-Cultural Smell Identification Test (CCSIT), calculated as the sum of the correct responses. The Beck Depression Inventory (BDI) was administered to assess the severity of depression. The CCSIT and BDI were measured within one month after the first clinic visit. Age at menopause was estimated using the age at the last menstrual cycle or at the time of bilateral oophorectomy. Lifetime exposure to estrogen (estrogen exposure length) was the sum of difference between age at menopause and menarche (reproductive years) and years of estrogen replacement therapy (ERT) use^12^. The estrogen deprivation length was the difference between age at PD onset and estrogen exposure length. The estrogen exposure ratio (E_ratio_) was calculated by dividing estrogen exposure length by total length of estrogen exposure and deprivation period; (age at menopause − age at menarche + ERT duration)/(age at PD onset − age at menarche), as illustrated in Fig. S1. According to E_ratio_, patients were classified into two groups: the low E_ratio_ group (n = 31) and the high E_ratio_ group (n = 31) based on the distribution. The exclusion criteria included atypical parkinsonism, drug-induced parkinsonism, and evidence of focal brain lesions, diffuse white matter hyperintensities, or multiple lacunes in the basal ganglia as determined by MRI. In addition, we performed supplementary analysis to evaluate age-related decline in DAT availability. Considering the mean age at PD onset in the low and high E_ratio_ groups (67.6 years and 55.9 years, respectively), female and male de novo PD patients whose mean age at PD onset was comparable to each E_ratio_ group were obtained from our previous cohort group^13^. The female PD group comprised of two subgroups whose mean age at PD onset was 68.0 years (PD-old [PD-O], n = 81) and 55.8 years (PD-young [PD-Y], n = 83). Similarly, the male PD group included two subgroups whose mean age at PD onset was 67.4 years (PD-O, n = 86) and 55.9 years (PD-Y, n = 82). Normal controls consisted of 25 healthy female individuals with no neurological diseases. Considering the difference of mean ages at PD onset in low and high E_ratio_ groups, we divided the control group (mean age, 61.4; n = 25) into two sub-groups whose mean ages were 67.4 years (control-old, n = 12) and 55.9 years (control-young, n = 13), which were comparable to mean ages at PD onset in the low and high E_ratio_ groups. Then, we also performed a comparative analysis of DAT availability between the control-young and high E_ratio_ groups as well as between the control-old and low E_ratio_ groups, respectively. These subjects were selected from our institute and underwent ^18^F-FP-CIT PET and brain MRI. E_ratio_ of the female PD-O and PD-Y groups was calculated by the information of reproductive factors obtained from the same questionnaire. This study was approved by the Yonsei University Severance Hospital ethical standards committee on human experimentation for experiments using human subjects and was therefore performed in accordance with the ethical standards laid down in the 1964 Declaration of Helsinki. Informed consent was waived because this study reviewed pre-existing data, which was approved by the Institutional Review Board of Yonsei University Severance Hospital (IRB No. 4-2014-0637).

### Longitudinal assessment of the changes in the levodopa equivalent dose

PD medications were prescribed after confirming decreased DAT availability in PP on the ^18^F-FP-CIT PET scans. The starting point of PD medications was at second clinic visit, which was within three months after the first visit. After diagnosis of PD, two movement disorder specialists (P.H.L. and Y.H.S.) adjusted the doses of PD medications for symptom control at 3–6-month intervals. At each visit, the doses of PD medication were checked, and the levodopa equivalent dose (LED) was calculated based on previously described methodology^14^.

### Quantitative analysis of the ^18^F-FP-CIT PET images

Image processing was performed using Statistical Parametric Mapping 12 (SPM12, Wellcome Department of Imaging Neuroscience, Institute of Neurology, London, England). For each patient, the reconstructed PET image was co-registered onto the corresponding MRI and normalized to the MNI152 template using normalization parameters defined from the corresponding MRI. Twelve volumes of interests (VOIs) of bilateral striatal sub-regions and one occipital VOI were drawn on the MNI152 template. The striatum was divided into right and left anterior/posterior caudate (AC/PC), anterior/ventral/posterior putamen (AP/VP/PP), and ventral striatum (VS) as previously described (Supplementary Methods)^13^. In the supplementary analysis performed by including our previous PD cohort group, we used the methodology for analyzing ^18^F-FP-CIT PET images that had been employed in a previous study^10^.

### Statistical analysis

Data are expressed as means ± standard deviations. Demographic and reproductive characteristics were compared using a student’s *t*-test for continuous variables, and Pearson’s χ^2^ test for categorical variables. An analysis of covariance was performed to compare DAT availability in striatal sub-regions after adjusting for age at PD onset and education duration. When comparing the specific to non-specific binding ratios (SNBRs) of the striatal sub-regions between groups, *P* values were further corrected for multiple comparisons using a false discovery rate method. The LED changes were estimated using a linear mixed model that included the following covariates: E_ratio_ group, age at PD onset, and baseline DAT availability in PP, time, and E_ratio_ group × time interaction. Pearson’s partial correlation analysis controlling age at PD onset and education duration was conducted to evaluate the relationship between E_ratio_ values and mean SNBRs on both sides of each striatal subregion. The statistical analyses were performed with SPSS (version 23.0; IBM Corporation, Armonk, NY, USA), and results with a two tailed *P* value of <0.05 were considered statistically significant.

## Results

### Demographic and reproductive characteristics

Demographic and reproductive characteristics of the low and high E_ratio_ groups and normal controls are provided in Table 1. As expected, PD patients in the low E_ratio_ group were older at PD onset (67.6 ± 1.3 vs 55.9 ± 1.3, *P* < 0.001) than those in the high E_ratio_ group. The education duration was significantly longer in control-old and control-young groups than in the low and high E_ratio_ groups. The low E_ratio_ group had higher UPDRS-motor score (*P* = 0.016) than the high E_ratio_ group. The proportion of PD subtypes did not differ between the two E_ratio_ groups. The CCSI score was significantly lower in the low E_ratio_ group than in the high E_ratio_ group. Regarding reproductive characteristics, age at menopause in the low E_ratio_ group was younger than in the high E_ratio_ group (*P* = 0.002), whereas age at menarche, the proportion of ERT use, and surgical menopause did not differ between the two E_ratio_ groups. The type of ERT formulation (estrogen-only or combined estrogen/progesterone therapy) was comparable between the two E_ratio_ groups. Additionally, the timing at initiation of ERT was comparable between the groups. The estrogen exposure length was significantly shorter in the low E_ratio_ group than in the high E_ratio_ group (*P* < 0.001), and the total length of estrogen exposure and deprivation period was significantly longer in the low E_ratio_ group than in the high E_ratio_ group (*P* < 0.001).

**Table 1:** Baseline demographic and reproductive characteristics.

Demographic characteristics	Low E_ratio_ group (N = 31)	High E_ratio_ group (N = 31)	Control-old (N = 12)	Control-young (N = 13)	*p* value (Low E_ratio_ vs High E_ratio_)	*p* value (Low E_ratio_ vs Control-old)	*p* value (High E_ratio_ vs Control-young)
Age, y	67.61 ± 1.32^a^	55.87 ± 1.34^a^	67.37 ± 5.47	55.88 ± 3.28	<0.001	0.926	0.995
Female, No. (%)	31 (100%)	31 (100%)	12 (100.0%)	13 (100.0%)	1.000	1.000	1.000
Education	8.46 ± 0.80	9.75 ± 0.80	14.00 ± 3.38	13.54 ± 2.44	0.287	0.001	0.004
Symptom duration before PD diagnosis, y	1.50 ± 1.41	1.80 ± 2.08			0.505		
UPDRS-III	22.54 ± 1.78	15.84 ± 1.61			0.016		
PD motor subtype, n (%)					0.685		
Tremor-dominant	3 (9.6%)	6 (19.3%)					
Akinetic-rigid	10 (32.3%)	11 (35.5%)					
Mixed	18 (58.1%)	14 (45.2%)					
CCSIT	6.54 ± 2.38	8.40 ± 2.36			0.004		
BDI	12.42 ± 7.68	12.83 ± 6.89			0.840		
K-MMSE	26.53 ± 0.45	27.22 ± 0.45			0.341		
Follow-up duration, y	2.70 ± 2.63	2.91 ± 2.73			0.755		
Total LED, mg	506.15 ± 208.67	350.36 ± 158.30			0.002		
Levodopa dose, mg	382.76 ± 193.66	184.82 ± 166.44			<0.001		
Reproductive characteristics							
Age at menarche, y	15.94 ± 1.71	15.70 ± 1.74			0.597		
Age at menopause, y	47.13 ± 5.33	51.37 ± 4.87			0.002		
Reproductive period, y	31.19 ± 5.46	35.67 ± 5.06			0.002		
Estrogen exposure length, y	31.51 ± 5.56	37.23 ± 4.32			<0.001		
Estrogen deprivation length, y	20.17 ± 7.69	2.93 ± 5.26			<0.001		
Estrogen exposure and deprivation length, y	51.68 ± 8.42	40.17 ± 6.32			<0.001		
E_ratio_	0.62 ± 0.10	0.92 ± 0.087			<0.001		
Ever use ERT, n (%)					0.740		
Yes	5 (16.1%)	6 (19.4%)					
No	26 (83.9%)	25 (80.6%)					
Types of ERT, n (%)					0.740		
Use of estrogen only	3 (60.0%)	3 (50.0%)					
Use of combined estrogen/progesterone	2 (40.0%)	3 (50.0%)					
Timing of ERT initiation, n (%)					0.455		
Within 10 years from menopause	4 (80.0%)	6 (100.0%)					
After 10 years from menopause	1 (20.0%)	0 (0.0%)					
Duration ERT use, y	0.86 ± 1.25	6.00 ± 5.03			0.010		
Surgical menopause, n (%)					0.159		
Yes	9 (29.0%)	4 (12.9%)					
No	22 (71.0%)	27 (87.1%)					

Data are expressed as mean ± standard deviation or number (percentage).

Abbreviations: UPDRS-III, Part III of the Unified Parkinson’s Disease Rating Scale; PD, Parkinson’s disease; K-MMSE, Korean version of Mini-Mental State Examination; CCSIT, Cross-Cultural Smell Identification Test; BDI, Beck Depression Inventory; LED, Levodopa equivalent dose; E_ratio_, Estrogen exposure ratio; ERT, Estrogen replacement therapy.

^a^Age at PD onset.

### SNBRs of PD subjects among the low and high E_ratio_ groups and control groups

**Figure 2:**
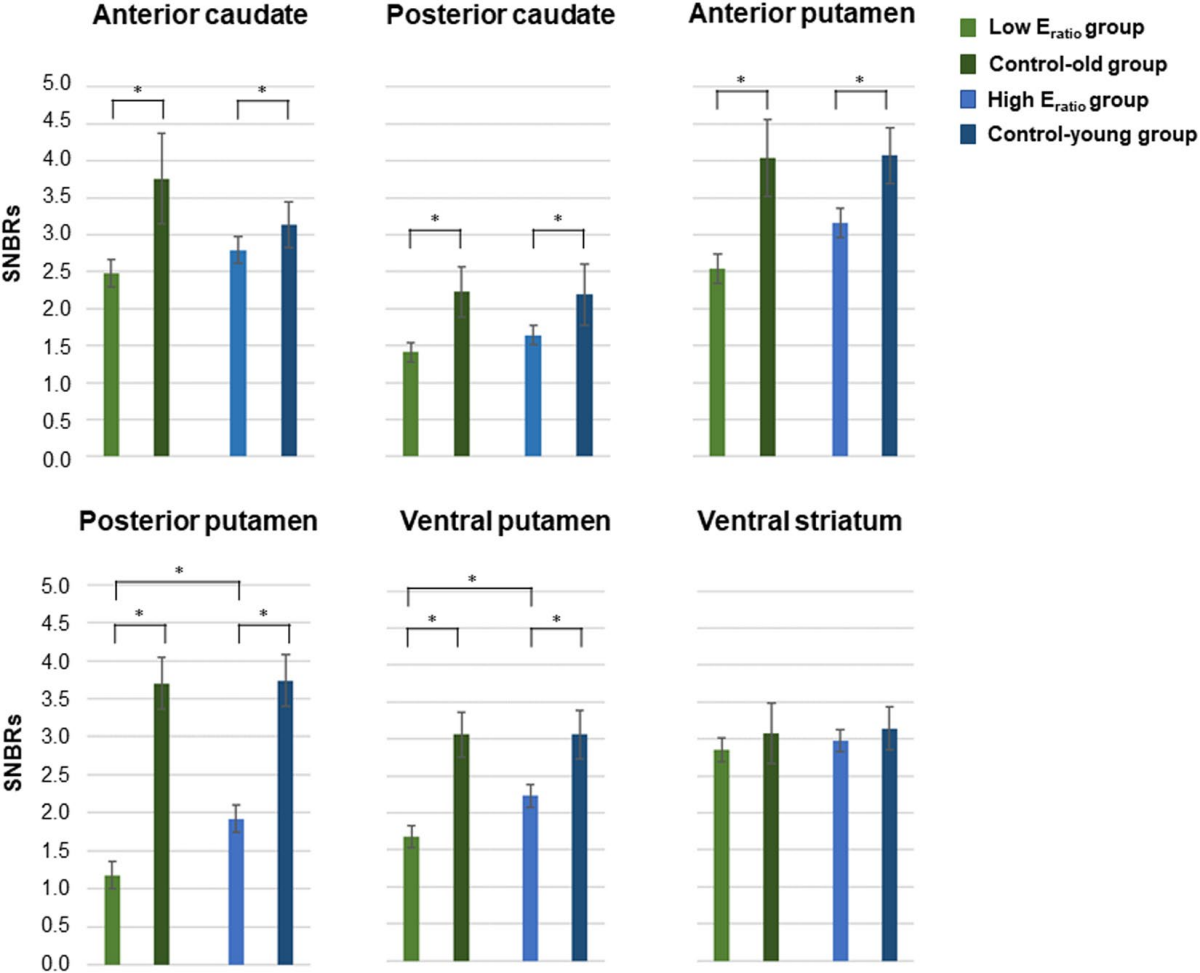
Mean SNBRs of both sides of each striatal sub-region in PD subjects among the low and high E_ratio_ groups and age- and sex-matched control groups. Mean SNBRs of both sides of each striatal sub-region in the low E_ratio_ group were significantly lower in the PP and the VP compared to the high E_ratio_ group. *P < 0.05. Abbreviations: SNBRs, Specific to non-specific binding ratios; PD, Parkinson’s disease; E_ratio_, estrogen exposure ratio; PP, posterior putamen; VP, ventral putamen.

SNBRs for striatal sub-regions in patients with the two E_ratio_ groups and control groups are presented in Table 2 and Fig. 2. When comparing DAT availabilities between the control-young and high E_ratio_ groups and between the control-old and low E_ratio_ groups, PD patients in the low or high E_ratio_ groups also showed significantly lower SNBRs in all sub-striatal regions, except for ventral striatum (Table 2). In a direct comparison between the two E_ratio_ groups, SNBRs in the low E_ratio_ group were significantly lower in PP (*P* = 0.024) and VP (*P* = 0.036) than in the high E_ratio_ group. This difference was pronounced in the more affected side of putamen than in the less affected side. However, SNBRs in other striatal sub-regions exhibited no significant difference between the two E_ratio_ groups. In addition, when classifying two groups not based on E_ratio_ but on estrogen exposure length itself, there was no significant difference in SNBRs on any striatal sub-regions between low and high E_ratio_ groups.

**Table 2:** SNBRs of PD subjects among the low and high estrogen exposure ratio groups and control groups.

Variables	Side	Low E_ratio_ group (N = 31)	Control-old (N = 12)	High E_ratio_ group (N = 31)	Control-young (N = 13)	*p* value^a^ (Low E_ratio_ vs High E_ratio_)	*p* value^a^ (Low E_ratio_ vs Control-old)	*p* value^a^ (High E_ratio_ vs Control-young)
Anterior caudate	More affected side	2.26 ± 0.21	3.62 ± 0.64	2.60 ± 0.20	3.72 ± 0.62	0.424	<0.001	0.002
	Less affected side	2.59 ± 0.21	3.68 ± 0.66	2.77 ± 0.20	3.80 ± 0.61	0.793	0.003	0.009
	Mean	2.48 ± 0.19	3.76 ± 0.61	2.79 ± 0.18	3.14 ± 0.31	0.334	0.001	0.003
Posterior caudate	More affected side	1.27 ± 0.14	2.19 ± 0.33	1.41 ± 0.13	2.14 ± 0.42	0.581	<0.001	0.002
	Less affected side	1.51 ± 0.15	2.27 ± 0.33	1.63 ± 0.14	2.24 ± 0.41	0.793	0.002	0.012
	Mean	1.41 ± 0.13	2.23 ± 0.34	1.64 ± 0.13	2.19 ± 0.41	0.332	0.001	0.003
Anterior putamen	More affected side	2.38 ± 0.23	4.00 ± 0.50	2.77 ± 0.22	3.01 ± 0.38	0.424	<0.001	<0.001
	Less affected side	2.69 ± 0.20	4.08 ± 0.53	3.24 ± 0.20	4.12 ± 0.39	0.140	0.002	<0.001
	Mean	2.54 ± 0.20	4.04 ± 0.52	3.16 ± 0.20	4.07 ± 0.38	0.096	0.001	<0.001
Posterior putamen	More affected side	0.89 ± 0.17	3.66 ± 0.34	1.57 ± 0.17	3.68 ± 0.33	0.027	<0.001	<0.001
	Less affected side	1.47 ± 0.17	3.74 ± 0.34	2.13 ± 0.17	3.80 ± 0.35	0.054	<0.001	<0.001
	Mean	1.18 ± 0.18	3.70 ± 0.34	1.92 ± 0.18	3.74 ± 0.34	0.024	<0.001	<0.001
Ventral putamen	More affected side	1.44 ± 0.15	2.99 ± 0.31	2.02 ± 0.15	3.01 ± 0.34	0.027	<0.001	<0.001
	Less affected side	1.90 ± 0.14	3.14 ± 0.31	2.40 ± 0.14	3.11 ± 0.34	0.057	<0.001	<0.001
	Mean	1.68 ± 0.15	3.06 ± 0.30	2.23 ± 0.15	3.06 ± 0.33	0.036	<0.001	<0.001
Ventral striatum	More affected side	2.54 ± 0.14	3.02 ± 0.42	2.73 ± 0.14	3.06 ± 0.32	0.424	0.042	0.114
	Less affected side	2.94 ± 0.16	3.15 ± 0.41	2.97 ± 0.15	3.21 ± 0.29	0.800	0.504	0.320
	Mean	2.85 ± 0.16	3.08 ± 0.41	2.98 ± 0.15	3.14 ± 0.29	0.497	0.462	0.461

Data are expressed as mean ± standard deviation.

Abbreviations: SNBRs, Specific to non-specific binding ratios; PD, Parkinson’s disease; E_ratio,_ Estrogen exposure ratio.

^a^False discovery rate-corrected *p* value of two group comparison.

### SNBRs of male and female PD-Y and PD-O patients

**Figure 3:**
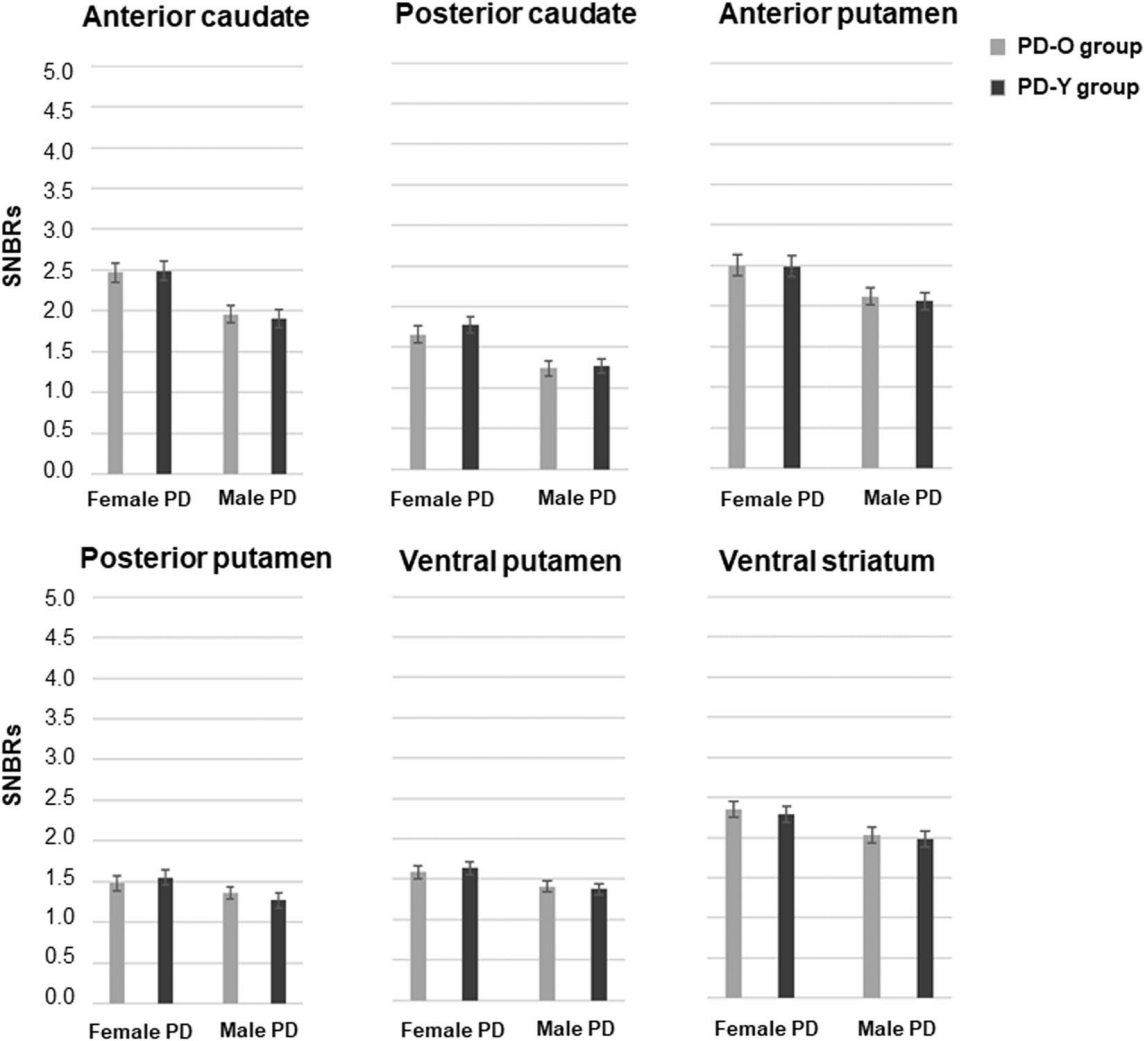
Mean SNBRs of both sides in striatal sub-regional activities in male and female patients with PD-Y and PD-O subgroups. Abbreviations: SNBRs, Specific to non-specific binding ratios; PD-O, PD-old; PD-Y, PD-young.

To evaluate whether aging influences striatal DAT availability, we further analyzed striatal SNBRs in PD-Y and PD-O subgroups of both sexes. Demographic characteristics of these subgroups are provided in Table S1. Except for education duration, clinical parameters including symptom duration, UPDRS-motor score, and K-MMSE score were comparable between male and female PD-Y and PD-O subgroups. Reproductive factors including the age at menarche, age at menopause, duration of ERT use, and estrogen exposure length exhibited no difference between the female PD-Y and PD-O subgroups. As expected, the E_ratio_ was higher in the female PD-Y group relative to the female PD-O group because of the difference in ages at PD onset. In contrast to sub-striatal DAT availabilities between the two E_ratio_ groups, SNBRs in all striatal sub-regions did not differ between male PD-Y and PD-O subgroups. Similarly, SNBRs in all striatal sub-regions did not differ between the PD-Y and PD-O subgroups in female PD patients. SNBRs of all striatal sub-regions in the PD-Y and PD-O subgroups in both sexes are presented in Fig. 3 and Table S2.

### Longitudinal changes in the LED during the follow-up period

The estimated monthly LED change was 10.9 in the low E_ratio_ group and 7.1 in the high E_ratio_ group, and there was significant interaction between the two groups and time in the linear mixed model, indicating that the monthly increase in the LED was significantly higher in the low E_ratio_ group than in the high E_ratio_ group (*P* = 0.001; Table 3).

**Table 3:** Longitudinal changes in LED across time in PD patients.

	Estimated slope (standard error)					
	Low E_ratio_ group	*p* value	High E_ratio_ group	*p* value	Difference	*p* value
Δ LED	10.9394 (0.8114)	<0.001	7.0584 (0.8880)	<0.001	3.8810 (1.2010)	0.001

The estimate (β) is the change in LED per month (Δ LED), i.e., positive value indicates the dose-up of PD medications. The effect of the PD subgroup (low estrogen exposure ratio group versus high estrogen exposure ratio group) on the change in LED across time was tested using the time x PD subgroup interaction term, and the low estrogen exposure ratio group exhibited a faster rate of dose-up of PD medications than the high estrogen exposure ratio group. Abbreviations: LED, levodopa-equivalent dose; PD, Parkinson’s disease; E_ratio,_ Estrogen exposure ratio.

### Relationship between E_ratio_ values and mean SNBRs on both sides of each striatal sub-region

E_ratio_ values showed significant positive correlation with mean SNBRs on both sides of PP, VP, and AP (PP, *P* = 0.012; VP, *P* = 0.020; AP, *P* = 0.048). SNBRs in other striatal sub-regions exhibited no significant correlation with E_ratio_ values (Table 4).

**Table 4:** Correlation analysis of the SNBRs and estrogen exposure ratio values.

Variables (mean of both sides)	E_ratio_ values (N = 62)	*p* value
Anterior caudate	0.219	0.112
Posterior caudate	0.205	0.138
Anterior putamen	0.271	0.048
Posterior putamen	0.339	0.012
Ventral putamen	0.316	0.020
Ventral striatum	0.149	0.283

Values are expressed as correlation coefficient.

Abbreviations: SNBRs, Specific to non-specific binding ratios; E_ratio,_ Estrogen exposure ratio.

## Discussion

The present study investigated whether estrogen exerts neuroprotective effect on nigrostriatal dopaminergic neurons in de novo PD patients using *in vivo* imaging. The major findings were as follows: (1) PD patients with higher E_ratio_ exhibited less severe parkinsonian motor symptoms than those with lower E_ratio_. (2) PD patients in the high E_ratio_ group had higher DAT availability in PP and VP than did those in the low E_ratio_ group. Although the age at PD diagnosis was significantly lower in the high E_ratio_ group relative to the low E_ratio_ group, aging did not influence sub-striatal DAT availability in male or female PD subjects. (3) The longitudinal increase in the estimated monthly LED change was lower in the high E_ratio_ group than in the low E_ratio_ group. (4) E_ratio_ values showed significant positive correlation with mean SNBRs on both sides of PP, VP, and AP.

In this study, we calculated E_ratio_ based on not only lifetime estrogen exposure period, but also estrogen deprivation period. Previous studies have reported conflicting results on neuroprotective effects of estrogen exposure length on PD risk^15,16^. Several epidemiological studies reported that longer fertile life length is associated with decreased risk of PD in postmenopausal women^17,18^. On the other hand, it has been suggested that estrogen deprivation period after menopause could be a pivotal factor in the influence of estrogen effects on neurodegenerative disorders^19^. Likewise, our study showed that simple estrogen exposure period did not influence on DAT availability in any striatal sub-regions. Therefore, E_ratio_ affected by both of the estrogen exposure and deprivation length could properly reflect estrogen-dependent pro-survival effect on dopaminergic neurons. Second, we performed a quantitative analysis of DAT availability using individual MRI co-registration to improve the accuracy of sub-striatal DAT patterns instead of a standard 18F-FP-CIT PET template acquired from healthy controls^10^.

The present study demonstrated that the high E_ratio_ group had higher DAT availability in PP and VP than did the low E_ratio_ group, and this difference was more pronounced in the more affected side than in the less affected side. It is well known that PP and VP, as the sensorimotor striatum^20^, receive projections from the dopaminergic neurons in the ventrolateral substantia nigra, and are connected to the primary motor and somatosensory cortices^21^. The results of the present study suggest that estrogen might exert a neuroprotective effect preferentially on nigrostriatal dopaminergic neurons that are primarily affected by PD pathology. Additionally, parkinsonian motor deficits were less severe in the high E_ratio_ group relative to the low E_ratio_ group, further supporting beneficial effect of estrogen on nigrostriatal dopaminergic system. Regarding pro-survival effects of estrogen, estrogen might suppress harmful inflammatory reactions, protect nigrostriatal dopaminergic neurons by regulating gene transcription, and target compensatory responses in surviving neurons to restore striatal functionality^22–24^.

Our data revealed an age discrepancy between the two E_ratio_ groups, since age at PD onset was included as a parameter when grouping patients. Since the end point of estrogen deprivation period is age at PD onset according to the formula of E_ratio_, the low E_ratio_ group with longer estrogen exposure and deprivation period had tendency to have older age at PD onset. This is the reason why the low E_ratio_ group appeared to have older age at PD onset in this study. However, our study showed less motor severity and less dopamine depletion of sensorimotor striatum in the high E_ratio_ group than in the low E_ratio_ group, even though symptom durations before PD diagnosis were comparable. Generally, DAT availability in the striatum is decreased with age in normal subjects, at the mean rate of 5.3% per decade^25,26^. Considering that the difference in ages at PD onset between low and high E_ratio_ groups is approximately ten years, our study showed 38.5% decline in mean SNBRs of posterior putamen and 19.3% decline in mean SNBRs of ventral putamen between the low and high E_ratio_ groups. These percentages of decline in DAT availability between the groups were much greater than aging-related decline. Furthermore, in patients with PD, the effect of age on DAT availability in the striatum seems to be inconsistent; some reports demonstrated that the aging effects were significantly smaller in PD patients than in healthy subjects^27^, whereas others reported no aging effect on DAT availability in the putamen^28–30^. Therefore, to clarify whether beneficial effect of estrogen on dopaminergic neurons in the high E_ratio_ group was secondary to the effect of age, we performed supplementary analysis by comparing DAT availability in PD-O and PD-Y groups of both sexes whose mean ages were comparable to each E_ratio_ group. However, DAT availabilities in PD-O and PD-Y groups of both sexes did not differ in any striatal sub-regions. Especially, all reproductive factors including the estrogen exposure length were comparable between the female PD-O and PD-Y groups except for the mean ages at PD onset. Therefore, the difference of the estrogen exposure and deprivation length between the female PD-O and PD-Y groups was caused solely by the mean ages at PD onset, and subsequently resulted in the difference of E_ratio_ values. Taken together, we can suggest that the effect of age alone does not lead to changes in DAT availabilities on the posterior and ventral putamen, but both estrogen exposure and deprivation length would have an impact on DAT availability selectively in the sensorimotor striatum.

Interestingly, the present study indicated that the longitudinal increase in the estimated monthly LED change during follow-up period was lower in the high E_ratio_ group than in the low E_ratio_ group. Although a longitudinal change in LED might not accurately reflect disease progression, LED appears to be indirectly associated with parkinsonian disability^31^. Regarding the prognostic role of estrogen, results of the present study may raise the possibility that estrogen can modulate longitudinal progression of parkinsonian motor deficits, although the difference in the longitudinal LED change may be secondary to the baseline difference in motor severity and sensorimotor striatal dopamine depletion.

There were several limitations in this study. First, this study was based on a questionnaire answered by postmenopausal participants who had to remember reproductive factors. Second, this study was based on a relatively small sample among a large cohort of more than 500 patients, which limits the generalization of our results possibly due to selection bias. However, we selected rigid inclusion and exclusion criteria to ascertain uniformity in clinical characteristics of enrolled patients, and strictly excluded those who could not remember their exact onset age of menarche and menopause to reduce recall bias as much as possible. In addition, because of small number of patients with surgical menopause or use of ERT, it was difficult to determine how these reproductive factors individually affect each striatal DAT availability. Especially, based on the difference in duration of ERT use between the two E_ratio_ groups, a further study focusing on patients with PD who received ERT would be needed to uncover this issue. Third, nigral neuron counts are not associated with striatal DAT availability in PD^32^. Therefore, caution should be exerted in the interpretation of our result that striatal DAT availability may reflect axonal dysfunction or DAT expression rather than the number of viable neurons.

In conclusion, the present study demonstrated that E_ratio_ was closely coupled with nigrostriatal DAT availability and longitudinal maintenance of dopaminergic medications in patients with postmenopausal de novo PD. These *in vivo* data provide indirect evidence that estrogen may have beneficial properties in PD.

## Supplementary information

Dataset 1

## Data Availability

For purposes of replicating procedures and results, any qualified investigator can request anonymized data after ethics clearance and approval by all authors.
