# Supplementary material for: Beneficial effect of estrogen on nigrostriatal dopaminergic neurons in drug-naïve postmenopausal Parkinson’s disease
Source: Sci Rep. 2019 Jul 19;9:10531. doi: 10.1038/s41598-019-47026-6 (PMC6642214; doi:10.1038/s41598-019-47026-6)

**Beneficial effect of estrogen on nigrostriatal dopaminergic neurons in drug-naïve postmenopausal Parkinson’s disease**

Yang Hyun Lee^1^, Jungho Cha^2^, Seok Jong Chung^1^, Han Soo Yoo^1^, Young H. Sohn^1^, Byoung Seok Ye^1^, Phil Hyu Lee^1, 3^

^1^Department of Neurology, Yonsei University College of Medicine, Seoul, South Korea

^2^ Memory and Aging Center, Department of Neurology, University of California San Francisco, San Francisco, CA, USA

^3^Severance Biomedical Science Institute, Yonsei University College of Medicine, Seoul, South Korea

**Supplementary Materials**

**^18^F-FP-CIT PET acquisition**

The ^18^F-FP-CIT PET scans were acquired using a GE PET-CT DSTe scanner (GE Discovery STE; GE Healthcare; Milwaukee, WI, USA), which obtains images with three-dimensional resolution of 2.3 mm full width at half maximum. All subjects were instructed to fast for ≥6 h before the PET scan and 5mCi (185 MBq) of ^18^F-FP-CIT was intravenously administered to the subjects. After 90 min post-injection, images were acquired for 20 min in the three-dimensional mode at 12 KVp and 380 mA.

**MRI acquisition**

All MRI scans were acquired using a Philips 3.0-T scanner (Philips Intera; Philips Medical System, Best, The Netherlands) with a SENSE head coil (SENSE factor = 2). A high-resolution T1-weighted MRI volume dataset was obtained from all subjects using a three-dimensional T1-TFE sequence configured with the following acquisition parameters: axial acquisition with a 224×256 matrix; 256×256 reconstructed matrix with182 slices; 220 mm field of view; 0.98×0.98×1.2 mm3 voxels; echo time, 4.6 ms; repetition time, 9.6 ms; flip angle, 8°; and slice gap, 0 mm.

**Quantitative analysis of the ^18^F-FP-CIT PET images**

The outer boundaries of the striatal sub-regions were visually determined by the characteristic dense grey signal of the striatum. The VOI for the ventral striatum was defined according to previously defined criteria^1^. The anterior commissure coronal plane was divided between the anterior and posterior caudate and between the anterior and posterior putamen. The boundary between the posterior and ventral putamen was the anterior-posterior commissure transaxial plane. DAT availability was calculated within individual grey matter regions as the following equation: (mean standardized uptake value of the striatal sub-region VOIs – mean standardized uptake value of the occipital VOI) / (mean standardized uptake of the occipital VOI)^2^.

**References**

1. Mawlawi, O. *et al.* Imaging human mesolimbic dopamine transmission with positron emission tomography: I. Accuracy and precision of D(2) receptor parameter measurements in ventral striatum. *Journal of cerebral blood flow and metabolism : official journal of the International Society of Cerebral Blood Flow and Metabolism* **21**, 1034-1057 (2001).

2. Innis, R. B. *et al.* Consensus nomenclature for in vivo imaging of reversibly binding radioligands. *Journal of cerebral blood flow and metabolism : official journal of the International Society of Cerebral Blood Flow and Metabolism* **27**, 1533-1539 (2007).

**Table S1.** Demographic characteristics between PD-O and PD-Y in female and male PD patients

|  | Female PD | | Male PD | | *p* value^a^ | *p* value^b^ |
| --- | --- | --- | --- | --- | --- | --- |
|  | PD-O (N = 81) | PD-Y (N = 83) | PD-O (N = 86) | PD-Y (N = 82) |  |  |
| Age at PD onset, y | 67.95 ± 2.61 | 55.76 ± 4.17 | 67.37 ± 2.74 | 55.92 ± 4.15 | <0.001 | <0.001 |
| Age at menarche, y | 15.15 ± 2.64 | 14.12 ± 2.65 |  |  | 0.083^c^ |  |
| Age at menopause, y | 51.05 ± 2.38 | 50.37 ± 2.60 |  |  | 0.218^c^ |  |
| Reproductive period, y | 35.90 ± 3.73 | 36.24 ± 3.54 |  |  | 0.672^c^ |  |
| Estrogen exposure period, y | 36.05 ± 3.70 | 36.40 ± 3.62 |  |  | 0.663^c^ |  |
| Estrogen deprivation period, y | 16.75 ± 2.66 | 5.23 ± 3.43 |  |  | <0.001^c^ |  |
| Estrogen exposure and deprivation period, y | 52.80 ± 2.56 | 41.64 ± 2.52 |  |  | <0.001^c^ |  |
| Ever use ERT, n (%) |  |  |  |  | 0.092^c^ |  |
| Yes | 9 (22.0%) | 4 (10.0%) |  |  |  |  |
| No | 32 (78.0%) | 36 (90.0%) |  |  |  |  |
| Duration ERT use, y | 0.15 ± 0.23 | 0.16 ± 0.42 |  |  | 0.872^c^ |  |
| Surgical menopause, n (%) |  |  |  |  | 1.000^c^ |  |
| Yes | 3 (7.3%) | 4 (10.0%) |  |  |  |  |
| No | 38 (92.7%) | 36 (90.0%) |  |  |  |  |
| Estrogen ratio | 0.68± 0.05 | 0.88 ± 0.08 |  |  | <0.001^c^ |  |
| Symptom duration before PD diagnosis, y | 1.35 ± 1.38 | 1.49 ± 1.77 | 1.62 ± 1.39 | 1.47 ± 1.22 | 0.304 | 0.455 |
| Education, y | 6.97 ± 4.46 | 9.64 ± 3.83 | 10.08 ± 4.71 | 11.82 ± 4.01 | <0.001 | 0.011 |
| UPDRS-III | 21.80 ± 8.06 | 20.13 ± 9.12 | 22.04 ± 8.78 | 20.88 ± 8.73 | 0.381 | 0.669 |
| K-MMSE | 26.38 ± 2.76 | 27.25 ± 2.16 | 26.69 ± 2.56 | 27.70 ± 1.95 | 0.856 | 0.598 |

Data are expressed as mean ± standard deviation.

Abbreviations: PD, Parkinson’s disease; UPDRS, Unified Parkinson’s Disease Rating Scale; K-MMSE, Korean-Mini Mental State Examination^;^ PD-O, PD-old; PD-Y, PD-young.

^a^Group comparison between PD-O and PD-Y in female PD patients.

^b^Group comparison between PD-O and PD-Y in male PD patients.

^c^115 patients with PD (58 with female PD-O and 57 with female PD-Y) underwent a detailed questionnaire on reproductive factors.

**Table S2.** Striatal Sub-regional Dopamine Transporter Activity between PD-O and PD-Y groups

| Variables | Side | Female PD | | Male PD | | *p* value^a^ | *p* value^b^ |
| --- | --- | --- | --- | --- | --- | --- | --- |
|  |  | PD-O (N = 81) | PD-Y (N = 83) | PD-O (N = 86) | PD-Y (N = 82) |  |  |
| Anterior caudate | More affected side | 2.34 ± 0.12 | 2.32 ± 0.12 | 1.85 ± 0.10 | 1.75 ± 0.11 | 0.980 | 0.584 |
|  | Less affected side | 2.59 ± 0.13 | 2.65 ± 0.12 | 2.07 ± 0.11 | 2.05 ± 0.11 | 0.783 | 0.943 |
|  | Mean | 2.47 ± 0.12 | 2.49 ± 0.12 | 1.96 ± 0.10 | 1.90 ± 0.11 | 0.913 | 0.761 |
| Posterior caudate | More affected side | 1.26 ± 0.09 | 1.31 ± 0.08 | 1.13 ± 0.09 | 1.16 ± 0.09 | 0.769 | 0.846 |
|  | Less affected side | 1.69 ± 0.11 | 1.79 ± 0.11 | 1.36 ± 0.09 | 1.37 ± 0.10 | 0.625 | 0.936 |
|  | Mean | 1.66 ± 0.10 | 1.78 ± 0.10 | 1.24 ± 0.09 | 1.27 ± 0.09 | 0.508 | 0.891 |
| Anterior putamen | More affected side | 2.31 ± 0.13 | 2.29 ± 0.12 | 1.98 ± 0.11 | 1.88 ± 0.11 | 0.921 | 0.620 |
|  | Less affected side | 2.68 ± 0.14 | 2.70 ± 0.13 | 2.27 ± 0.12 | 2.25 ± 0.12 | 0.948 | 0.924 |
|  | Mean | 2.50 ± 0.13 | 2.49 ± 0.13 | 2.12 ± 0.11 | 2.06 ± 0.11 | 0.989 | 0.771 |
| Posterior putamen | More affected side | 1.25 ± 0.09 | 1.29 ± 0.08 | 1.17 ± 0.07 | 1.04 ± 0.07 | 0.777 | 0.294 |
|  | Less affected side | 1.69 ± 0.11 | 1.77 ± 0.11 | 1.55 ± 0.10 | 1.50 ± 0.10 | 0.691 | 0.806 |
|  | Mean | 1.48 ± 0.09 | 1.55 ± 0.09 | 1.36 ± 0.08 | 1.27 ± 0.09 | 0.673 | 0.203 |
| Ventral putamen | More affected side | 1.44 ± 0.08 | 1.45 ± 0.07 | 1.27 ± 0.06 | 1.18 ± 0.06 | 0.930 | 0.436 |
|  | Less affected side | 1.75 ± 0.10 | 1.83 ± 0.10 | 1.54 ± 0.09 | 1.58 ± 0.09 | 0.634 | 0.817 |
|  | Mean | 1.59 ± 0.08 | 1.64 ± 0.08 | 1.41 ± 0.07 | 1.38 ± 0.07 | 0.751 | 0.845 |
| Ventral striatum | More affected side | 2.23 ± 0.10 | 2.17 ± 0.10 | 1.91 ± 0.10 | 1.86 ± 0.10 | 0.750 | 0.762 |
|  | Less affected side | 2.48 ± 0.11 | 2.41 ± 0.11 | 2.15 ± 0.10 | 2.11 ± 0.11 | 0.735 | 0.821 |
|  | Mean | 2.35 ± 0.10 | 2.29 ± 0.10 | 2.03 ± 0.10 | 1.98 ± 0.10 | 0.739 | 0.789 |

Data are expressed as mean ± standard deviation.

Abbreviations: PD, Parkinson’s disease; PD-O, PD-old; PD-Y, PD-young.

^a^Group comparison between PD-O and PD-Y groups in female PD patients

^b^Group comparison between PD-O and PD-Y groups in male PD patients

**Figure S1.** Schematic representation for calculation of estrogen exposure ratio. Abbreviations: ERT, Estrogen replacement therapy; PD, Parkinson’s disease


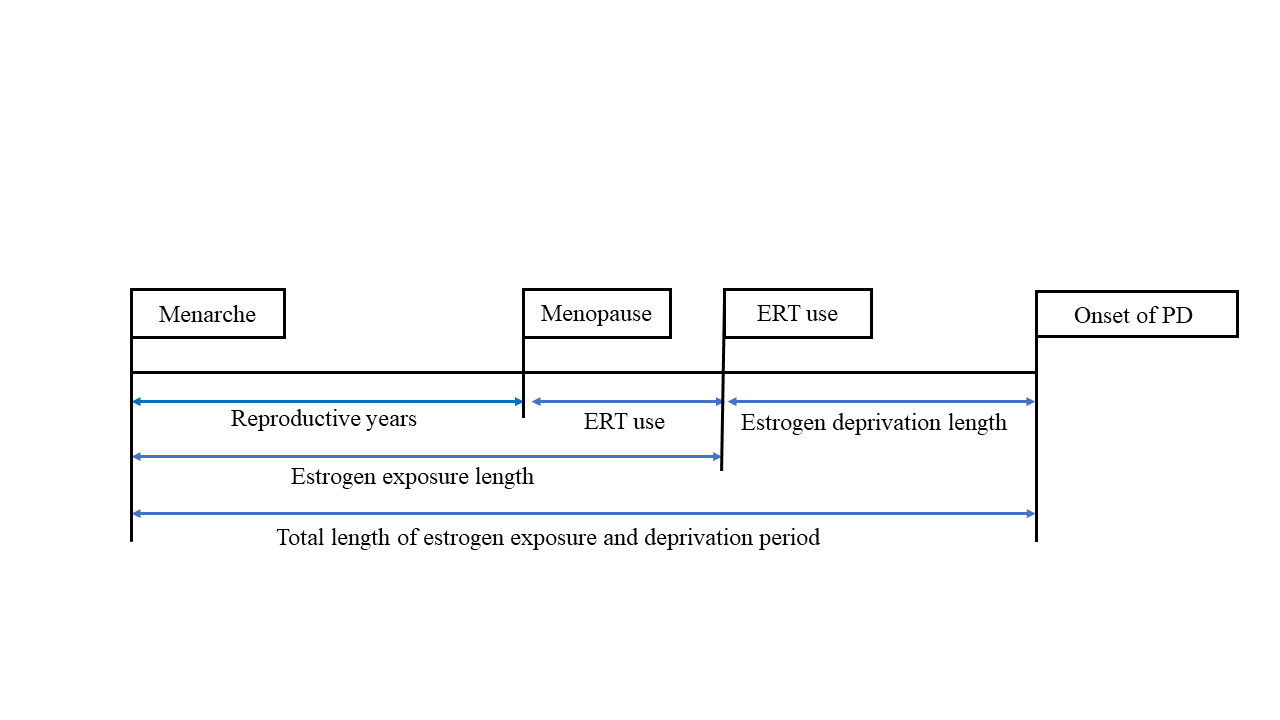

Supplement: Supplementary file 1 — Dataset 1 [file 41598_2019_47026_MOESM1_ESM.docx]
